# Rapid MALDI-TOF Mass Spectrometry Strain Typing during a Large Outbreak of Shiga-Toxigenic *Escherichia coli*

**DOI:** 10.1371/journal.pone.0101924

**Published:** 2014-07-08

**Authors:** Martin Christner, Maria Trusch, Holger Rohde, Marcel Kwiatkowski, Hartmut Schlüter, Manuel Wolters, Martin Aepfelbacher, Moritz Hentschke

**Affiliations:** 1 Department of Medical Microbiology, Virology and Hygiene, University Medical Center Hamburg-Eppendorf, Hamburg, Germany; 2 Institute of Organic Chemistry, Mass Spectrometry Facility, University of Hamburg, Hamburg, Germany; 3 Department of Clinical Chemistry, Mass Spectrometric Proteomics, University Medical Center Hamburg-Eppendorf, Hamburg, Germany; University of Lausanne, Switzerland

## Abstract

**Background:**

In 2011 northern Germany experienced a large outbreak of Shiga-Toxigenic *Escherichia coli* O104:H4. The large amount of samples sent to microbiology laboratories for epidemiological assessment highlighted the importance of fast and inexpensive typing procedures. We have therefore evaluated the applicability of a MALDI-TOF mass spectrometry based strategy for outbreak strain identification.

**Methods:**

Specific peaks in the outbreak strain’s spectrum were identified by comparative analysis of archived pre-outbreak spectra that had been acquired for routine species-level identification. Proteins underlying these discriminatory peaks were identified by liquid chromatography tandem mass spectrometry and validated against publicly available databases. The resulting typing scheme was evaluated against PCR genotyping with 294 *E. coli* isolates from clinical samples collected during the outbreak.

**Results:**

Comparative spectrum analysis revealed two characteristic peaks at m/z 6711 and m/z 10883. The underlying proteins were found to be of low prevalence among genome sequenced *E. coli* strains. Marker peak detection correctly classified 292 of 293 study isolates, including all 104 outbreak isolates.

**Conclusions:**

MALDI-TOF mass spectrometry allowed for reliable outbreak strain identification during a large outbreak of Shiga-Toxigenic *E. coli*. The applied typing strategy could probably be adapted to other typing tasks and might facilitate epidemiological surveys as part of the routine pathogen identification workflow.

## Introduction

Effective tracking of highly resistant or hypervirulent pathogens requires the assessment of clonal relationship among clinical isolates. Due to high costs and long turnaround times, established nucleic acid based typing methods of sufficient discriminatory power, such as pulsed field gel electrophoresis (PFGE) or multilocus sequence typing (MLST), are primarily used for retrospective analyses and small samples sizes. More rapid and affordable alternatives, such as single gene sequencing or PCR-based genotyping, are only established for certain genera or widely distributed clones. In a routine setting, outbreak detection and surveillance still heavily rely upon phenotypic tests of limited discriminatory power, such as biochemical or antibiotic resistance profiling.

In recent years, matrix assisted laser desorption/ionization time of flight mass spectrometry (MALDI-TOF MS) has been established for culture based pathogen identification in many clinical microbiology laboratories [1]–[2]. The technique is based upon the analysis of whole cell mass spectra representing dozens of microbial proteins as peaks with an exactly determinable mass to charge (m/z) ratio. The observed degree of molecular mass conservation among these proteins renders spectral similarity a suitable marker of phylogenetic kinship and enables current commercially available fingerprinting systems to reliably infer species identity of unknown isolates from whole spectrum similarity comparisons with reference spectra [3]. Although these measures have sporadically been applied for subspecies differentiation [4]–[6], their use for epidemiological purpose is impeded by the lack of suitable reference spectrum collections, the complexity of threshold setting and limitations in discriminatory power. In order to improve the phylogenetic resolution of whole cell mass spectrometry, weighted pattern matching algorithms and biomarker based strategies have been proposed. By focusing the analysis on a small subset of discriminatory peaks, these measures theoretically facilitate reliable detection of single peak differences between strains. Their application already allowed for successful discrimination between well recognized subtypes of *Clostridium difficile*, *E. coli*, *Salmonella enterica* and *Yersinia enterocolitica* [7]–[10]. Two proof-of-principle studies identified characteristic marker peak combinations for certain lineages of methicillin resistant *Staphylococcus aureus*, thus highlighting the technique’s capability for epidemiological purpose [11]–[12]. As a major drawback, these approaches relied upon the analysis of purpose built reference strain collections for biomarker discovery which reduces flexibility and aggravates external validation in the absence of publicly accessible spectrum databases.

The present study proposes a general applicable workflow for the development of biomarker based MALDI-TOF MS typing schemes with recourse to locally and publicly available data and describes its successful implementation during 2011’s large outbreak of Shiga-Toxigenic *E. coli* (STEC) in northern Germany [13].

## Methods

### Samples and study design

A marker peak based strategy for MALDI-TOF MS strain typing was evaluated during a large STEC outbreak in spring/summer 2011 [13]. Outbreak strain specific spectral biomarkers were discovered by comparison of reference spectra from STEC outbreak isolate TY-2482 (ATCC BAA-2326, NCBI Taxonomy ID 1038844, BioProject accession PRJNA67657) [14] to a random selection of archived pre-outbreak spectra, which had previously been acquired for routine MALDI-TOF MS based species-level identification in our clinical microbiology laboratory. Proteins underlying the discovered discriminatory peaks were identified by liquid chromatography tandem mass spectrometry (LC-MS/MS). Specificity was confirmed with available nucleic acid and protein databases. Validated marker peaks were used to classify prospectively acquired *E. coli* spectra from stool, rectal swab and urine isolates, recovered in our clinical microbiology laboratory between June and August 2011. Results from marker peak based mass spectrometry typing were compared to reference classification by PCR genotyping and MLST. In addition, various whole spectrum similarity measures were applied to our study spectra to test their applicability for typing purpose and to assess the overall spectral variability among endemic *E. coli* isolates.

### MALDI-TOF mass spectrometry

Study isolates were prepared for mass spectrometry measurements from Columbia blood agar cultures after 16 to 24 hours of incubation [15]. For formic acid extraction, colony material was suspended in 300 µl distilled water, mixed with 900 µl ethanol, and centrifuged for 2 min at 13,000×g in a tabletop microcentrifuge. Supernatant was discarded and residual ethanol removed after repeated centrifugation. The pellet was resuspended in 35 µl 70% formic acid and mixed with 35 µl acetonitrile. After a final centrifugation, 1 µl aliquots of the supernatant were spotted in triplicate on a ground steel target and air dried at room temperature. Sample spots were overlain with 1.5 µl matrix solution (saturated solution of α-cyano-4-hydroxy cinnamic acid in 50% acetonitrile with 2.5% trifluoroacetic acid) and air dried at room temperature.

For direct sample deposition, colony material was collected with a wooden toothpick, spotted in triplicate on a ground steel target and overlain with 1.5 µl matrix solution as described above. In addition to the samples, preparations of a mixture of *E. coli* strain DH5α proteins (Bacterial Protein Standard, Bruker Daltonics) were spotted on each target for instrument calibration. Spectra were acquired with a Microflex LT mass spectrometer operated by the MALDI-Biotyper automation control (Bruker Daltonics) using recommended settings for bacterial species identification (linear positive mode, 20-Hz laser frequency, 20-kV acceleration voltage, 18.5-kV IS2 voltage, 250 ns extraction delay, and 2,000 to 20,000 m/z range).

Archived pre-outbreak spectra from routine species level identification had been acquired as single spectra by direct sample deposition as describe above.

### Spectrum processing

Spectra were internally calibrated in flex analysis 2.1 (Bruker Daltonics) with known m/z-values of highly conserved ribosomal proteins (RL36, RS32, RS34, methylated RS33, RL29 and RS19) and exported as tab-separated text files. Further processing was performed with the MALDIquant package 1.7 for R 2.15.2 [16]–[17]. Optimal parameter settings for smoothing, baseline correction and peak detection were empirically determined by the analysis of TY-2482 reference spectra. Nine formic acid extraction and nine direct sample deposition replicate spectra from three independent cultures were processed with a range of different values for each processing parameter (smoothing: moving average with half window size 2, 4, 6, 8, 10, 12 and 16; baseline correction: Statistics-sensitive Non-linear Iterative Peak-clipping algorithm (SNIP) with half window size 25, 50, 75, 100 and 200; peak detection: median absolute deviation (MAD) with half window size 4, 8, 12, 16 and 20 and signal to noise ratio threshold 2, 3, 4, 6, 8, 10 and 12). For each parameter combination and sample preparation method, the number and proportion of reliably detectable peaks (peaks with a detection frequency >7/9) were determined. The combination of parameter values yielding the largest product of these numbers for both sample preparation methods was used for all subsequent analyses.

For MALDI Biotyper analyses, calibrated raw spectra were processed with MALDI Biotyper 3.0 (Bruker Daltonics). Default values for bacterial species level identification were used for smoothing (Savitsky-Golay with frame size 25), baseline correction (multipolygon with search window 5 and number of runs 2) normalization (maximum norm) and peak detection (spectra differentiation with signal to noise ratio 3 and threshold 0.001).

M/z-tolerance for calibration and peak detection was set to 400 ppm as suggested by the distribution of m/z-positions of eight reference peaks among the 2×9 TY-2482 reference spectra (3×SD = 334 ppm).

### Biomarker peak discovery

Outbreak strain specific marker peaks were discovered by automated comparison of outbreak isolate TY-2482 reference spectra to a random selection of archived pre-outbreak *E. coli* spectra. Peaklists from 3×3 formic acid extraction and 3×3 direct sample deposition TY-2482 reference spectra were filtered for peak occurerrence frequency (>7/9) and merged into combined peaklists for the two sample preparation methods using MALDIquant’s filterPeaks and mergeMassPeaks functions. For each peak that appeared in both of these sample preparation method specific peaklists, the occurrence rate within the population of endemic isolates was estimated by the analysis of 150 pre-outbreak *E. coli* spectra (identifications score ≥2.3) from the archive of the MALDI-Biotyper MS fingerprinting system (Bruker Daltonics) used for routine species identification in our clinical microbiology laboratory. Pre-outbreak spectra were processed as described above and searched for the presence of TY-2482 peaks using an m/z tolerance of 400 ppm. Peaks within the lowest quintile of the occurrence rate distribution were visually examined to exclude artifact signals. From the remaining peaks, a set of outbreak strain specific marker peaks was chosen based on peak occurrence rates and signal to noise ratios.

### Biomarker protein identification by molecular weight matching

Presumptive identification of the proteins represented by discriminatory peaks was pursued by molecular weight matching [9] against the protein databases at the European Bioinformatics Institute (EBI) and the National Center for Biotechnology Information (NCBI) using TagIdent [18] or suitable ENTREZ queries. The molecular weight of biomarker proteins was derived from the corresponding marker peak’s m/z ratio considering simple protonation (m/z−1), double protonation (2×m/z−2) and methionine loss (m/z−1+132.2 and 2×m/z−2+132.2). Search tolerance was set to 400 ppm. Queries were limited to *E. coli* O104:H4 (Taxonomy ID 1038927) as the source organism.

### Biomarker protein identification by LC-MS/MS

Identification of biomarker peaks was performed by LC-MS/MS after protein purification from TY-2482 formic acid extracts by isoelectric focusing and reversed phase chromatography. Fivehundred µl formic acid extracts of bacterial overnight cultures on Columbia blood agar were prepared as described above. Buffer was changed towards offgel sample buffer (20% methanol, 1% GE Healthcare IPG buffer pH 3–10) by ultrafiltration in Amicon Ultra-4 filter devices (Millipore) with a 3 kDa molecular weight cut-off. Sample volume was adjusted to 3.6 ml. Isoelectric focusing was performed on a 3100 Offgel Fractionator (Agilent Technologies) within a linear gradient from pH 3 to 10 subdivided into 24 fractions with a maximum current of 50 µA for a total of 50 kVh. Aliquots of all fractions were mixed 1∶1 with matrix solution and analyzed by MALDI-TOF mass spectrometry (MS) on an ultrafleXtreme instrument (Bruker Daltonics). Fractions containing the protein of interest were vacuum dried, resolved in 1 ml of RPC buffer A (0.1% trifluoroacetic acid) and subjected to further separation by reversed phase chromatography. Nine-hundred µl sample were loaded on a Poroshell 300SB-C8 2.1 mm×10 cm column (Agilent Technologies) at a concentration of 2% RPC buffer B (100% acetonitrile) with a flow rate of 1 ml/min. Proteins were eluted in 1 ml fractions using a linear gradient from 2 to 70% RPC buffer B within 60 min. All fractions were vacuum-dried and resolved in 10 µl of 30% acetonitrile and 0.1% trifluoroacetic acid prior to analysis by MALDI-TOF MS. Fractions containing the proteins of interest were dried and dissolved in 25 µl of 6 M Urea. After addition of 0.7 µl 100 mM dithiothreitol in digestion buffer (100 mM NaHCO3, pH 8.3), samples were incubated at 60°C for 10 minutes to reduce disulfide bridges. Free cysteine residues were blocked by incubation for 30 min in the dark after addition of 0.7 µl 300 mM iodoacetamide in digestion buffer. Trypsin digestion was performed for 16 hours after addition at 37°C after addition of 225 µl digestion buffer and 4 µl of 0.25 mg/ml sequencing grade modified trypsin (Promega). The reaction was stopped by the addition of formic acid to a final pH of 3. Identification of tryptic peptides was performed on an 1100 LC/MSD-trap XCT series mass spectrometer equipped with a ChipCube electrospray ionization system and a large capacity chip (Agilent Technologies). 8 µL sample were loaded onto the enrichment column at a flow rate of 4 µl/min with the mix of mobile phase A (0.2% formic acid in H2O) and mobile phase B (100% acetonitrile) at a ratio 98∶2. The liquid chromatography (LC) gradient was delivered with a flow rate of 400 nl/min. Tryptic peptides were eluted using a linear gradient of 2 to 40% mobile phase B in 40 min. MS experiments were performed with a 300 to 2000 m/z scan range, positive polarity and a capillary voltage of −1800 V. Flow rate and temperature of the drying gas were 4 l/min and 325°C, respectively. The MS/MS experiments were carried out in autoMS/MS mode using a window of 4 Da for precursor ion selection and an absolute intensity threshold of 10,000. After 3 MS/MS spectra, the precursor ions were excluded from fragmentation for one minute. The generic files for database searching were generated by Data Analysis software version 3.4. A signal to noise ratio threshold of 5 was applied for precursor ion selection and the absolute number of compounds was restricted to 1000 per run. Protein identification was performed with Mascot online search on www.matrixscience.com using the default significance threshold of 0.05 [19]. MS/MS datasets were used to search the ‘Bacteria’ subset of NCBI’s nr database.

### Typing scheme validation against publicly available sequence data

Occurrence frequencies of the identified biomarker proteins among *E. coli* strains was estimated by comparison of the respective protein encoding sequences against all NCBI refseq_genomic database sequences beneath the *E. coli* taxonomic level (TaxID: 562) [20]. The number of database matches translating into proteins of the correct molecular weight was related to the total number of deposited whole genome or plasmid sequences.

### Marker peak based isolate classification by MALDI-TOF MS

Study isolates were classified as outbreak related or non outbreak related based on the presence or absence of the predefined marker peaks. The m/z-tolerance for establishment of marker peak presence was set to 400 ppm. Peak detection in only one of three replicate spectra required confirmation by visual spectrum examination and repeated measurement.

### Reference classification by PCR genotyping and MLST

Reference classification was based upon PCR detection of characteristic genetic features of the outbreak strain [21]. DNA was prepared from freshly grown overnight cultures by suspending 10 µl loops of colony material in 300 µl TE buffer and incubating for 10 minutes at 95°C with subsequent centrifugation. PCR reactions targeting *stx2* (Shiga-toxin), *terD* (part of a tellurium resistance gene cluster), *rfbO104* (part of the O104 lipopolysaccharide antigen biosynthesis gene cluster), *flicH4* (part of the H4 flagellar antigen biosynthesis gene cluster) and *aggC* (part of the aggregative adherence fimbria I biosynthesis gene cluster) were performed as described previously [22]–[23]. Isolates that tested positive for all five marker genes were classified as outbreak related. Considering the potential loss of the mobile genetic markers *stx2* and *aggC* [24], isolates lacking one of these markers were also classified as outbreak related if they shared the outbreak strain’s MLST profile [25]. All other isolates were classified as non outbreak related.

### Genotype correlation of MALDI-TOF phenotypes

Genotype correlation of the observed MALDI-TOF phenotypes was assessed by PCR testing of study isolates for biomarker protein encoding genes, allele sequencing in case of discrepancies between PCR testing and MALDI-TOF classification and plasmid restriction mapping. PCR testing for biomarker protein encoding genes was done with primer pairs located within the coding region of the respective genes. Additional primers, located up- and downstream the coding region, were utilized to amplify DNA for allele sequencing (table 1). All amplification reactions were performed in total volumes of 25 µl containing 12.5 µl REDTaq Ready Mix (Sigma-Aldrich), 1 pmol forward and reverse primers and 2 µl template (prepared as for PCR genotyping) with 35 cycles of denaturation at 94°C for 30 s, annealing at 55°C for 60 s and extension at 72°C for 120 s. Sanger sequencing of purified PCR products was performed by a commercial supplier (MWG Eurofins). Plasmid DNA from selected study isolates was prepared from 5 ml overnight cultures in Luria Bertani broth (LB) using Qiagen’s QIAprep spin Miniprep kit. Transformation into chemically competent *E. coli* TOP10 (life technologies) was performed according to manufacturer’s instructions. ChromID ESBL agar (Biomérieux) was used as a selective medium to screen transformants for the presence of ESBL plasmids. Plasmid DNA for restriction mapping was prepared from 50 ml overnight cultures of the transformants in Luria Bertani broth (LB) using Qiagen’s QIAprep spin Miniprep kit with four times the recommended volumes of buffers P1, P2 and N3 to account for the increased volume of starting material. Restriction digestion was performed with DraI and HindIII FastDigest Enzymes (Fermentas) according to manufacturer’s instructions.

**Table 1:** Oligonucleotides for biomarker gene detection and sequencing.

Oligonucleotide	Sequence (5′-3′)	Amplicon [bp]^a^
mp6711-detection_f	CTATCGCAGAACGTCTTGAGC	144
mp6711-detection_r	CGGTAGCCTGCTTAATCTGC	
mp6711-sequencing_f	CCGCAGCATGAGGATAAACT	360
mp6711-sequencing_r	ACTTCAAGCGCACCTTTCTT	
mp10883-detection_f	CCCTGCCAGCGATTTCAGCA	168
mp10883-detection_r	GCGCTACCATGCCTTTCGCA	
mp10883-sequencing_f	CAGGGGGCATTTTTATCAGA	510
mp10883-sequencing_r	CCGAAATTAATCGGGTTTGA	

a In *E. coli* TY-2482.

### Isolate classification by whole spectrum similarity

Isolate classification by whole spectrum similarity was based on distances between study isolates’ replicate spectra and reference spectra from outbreak isolate TY-2482. A selection of binary, metric and correlation based distance measures were employed with formic acid extraction and direct sample deposition spectra and evaluated with receiver operating characteristic (ROC) curves. Performance was compared with DeLong’s test (paired curves) or bootstrapping (unpaired curves) for differences in the area under the ROC curve (AUC) using the pROC package 1.6 for R [26]. A significance level of 0.05 was used without adjustment for multiple testing.

Jaccard, Mountford, Braun-Blanquet, Simpson, Ochiai (binary), Euclidean, Bhjattacharyya, Divergence, Manhattan, Canberra (metric) and Pearson (correlation) distances were determined in R using the proxy package version 0.4–10. Spectral distance to TY-2482 was calculated for each study isolate and sample preparation method as the lowest distance from all 3×9 pairwise comparisons between this isolate’s replicate spectra and the corresponding 3×3 TY-2482 replicate spectra.

The problem of prospective treshold setting was adressed by computing bootstrap estimates (n = 1000) of 95% confidence intervals for thresholds derived from the distribution of pairwise whole spectrum similarity (mean+2.3×SD) among three to 25 outbreak isolates.

MALDI-Biotyper similarity scores were determined with MALDI-Biotyper 3.0. Study spectra were individually matched against the corresponding sample preparation method specific TY-2482 reference profile (main spectrum, MSP) using default parameter settings for bacterial species level identification [3]. MSPs had been created from the respective 3×3 TY-2482 replicate spectra using default parameter settings for MSP creation (maximum mass error for each single spectrum 2000, mass error for the MSP 200, peak frequency minimum 25%, maximum peak number 70). Biotyper similarity to TY-2482 was defined as the highest Biotyper score obtained from the matching of three replicate spectra.

### Assessment of spectral variability among endemic *E. coli* isolates

Spectral variability among endemic *E. coli* isolates was estimated by pairwise whole spectrum similarity comparisons between non-outbreak study isolates. Spectral distance was calculated in R as the lowest Jaccard distance obtained from the 3×3 possible pairwise comparisons between two isolates’ formic acid extraction triplicate spectra. A threshold indicating spectral identity (mean+2×SD) was derived from the distribution of spectral distances among replicate spectra after removal of outliers (distance <Q1–1.5×IQR or >Q3+1.5×IQR). This threshold was applied to complete linkage hierarchical clustering to calculate Simpson’s diversity index as a measure of spectral variability. In addition, pairs of triplicate spectra below the 5th percentile of the spectral distance distribution were manually checked for qualitative differences in the presence of detectable non-artifact peaks.

## Results

### Optimization of spectrum processing parameters

**Figure 1:**
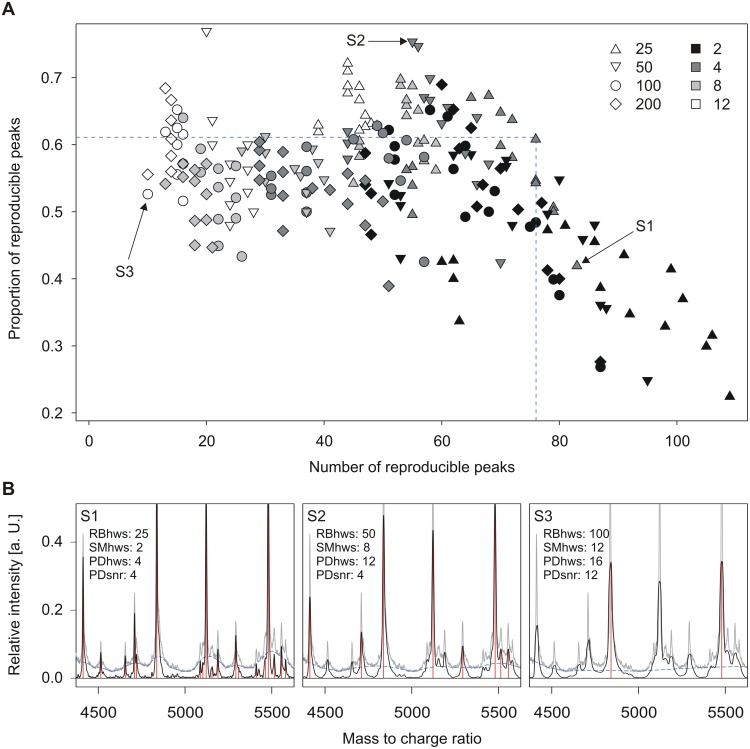
Effect of spectrum processing parameters on peak reproducibility. Number and proportion of reproducible peaks in TY-2482 formic acid extraction replicate spectra as a function of spectrum processing parameters (A). Half window sizes for SNIP baseline correction and signal to noise ratio thresholds for peak detection are represented by symbol and fill colour, respectively. For each combination, 16 variants representing different half window sizes for smoothing (2, 4, 8, 12) and peak detection (4, 8, 12, 16) are shown. Dashed lines mark the parameter combination employed for all subsequent analyses. Representative spectra from extreme positions of the parameter space (arrows) are shown in detail (B).

Processing parameter settings considerably affected peak reproducibility in a test set of 2×3×3 TY-2482 replicate spectra. The parameter settings selected for all subsequent spectrum analyses (smoothing: moving average with half window size 4; baseline correction: SNIP with half window size 25; peak detection: MAD with half window size 12 and signal to noise ratio threshold 4) represented the best compromise with respect to the number and proportion of reproducible peaks resulting from the application of these settings to the TY-2482 test spectra (figure 1).

### Biomarker discovery and identification

**Figure 2:**
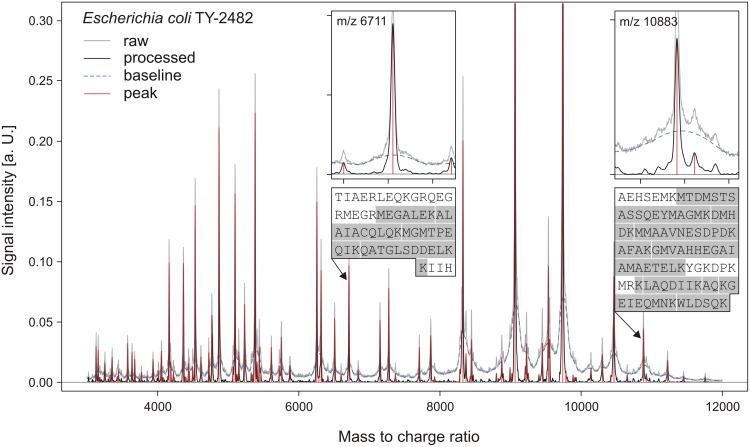
MALDI-TOF mass spectrum of *E. coli* outbreak isolate TY-2482. Representative whole cell MALDI-TOF mass spectrum of the Shiga-Toxigenic *E. coli* outbreak isolate TY-2482 acquired after formic acid extraction. Inlays show enlarged views of outbreak strain specific marker peaks and the amino acid sequence of the corresponding proteins. Peptides identified by LC-MS/MS are indicated by a gray background. The tick mark interval in the enlarged peak views is set to 100.

About 90 peaks within the 3000 to 12000 m/z range could be detected in whole cell MALDI-TOF mass spectra of the STEC O104:H4 outbreak strain TY-2482 acquired with standard instrument settings for microbial identification (figure 2). Sixty of these peaks were classified as reliably detectable based on signal to noise ratio and assay to assay reproducibility. Comparison to 150 archived pre-outbreak *E. coli* spectra identified six peaks (m/z 3445, m/z 6711, m/z 6842, m/z 9450, m/z10883, m/z 10922) with low occurrence rate (<0.1) in these routinely acquired direct deposition spectra from endemic isolates. Two peaks (m/z 9450 and m/z 10922) were correlated with higher prevalent ‘sibling peaks’ (m/z 4725 and m/z 5460) that probably represented a differently charged version of the same underlying protein. Based on estimated occurrence rates (0.0% and 4.7%) and signal to noise ratios (9.8 and 29.8), the peaks at m/z 6711 and m/z 10883 were chosen as outbreak strain biomarkers.

The corresponding proteins could be identified by LC-MS/MS after purification from bacterial formic acid extracts with electrophoretic and chromatographic methods. The peak at m/z 6711 represents a 61 amino acids protein with a calculated molecular weight of 6709.8 Da, homologous to the C-terminal part of the predicted transposase YdgA (GenPept accession YP_004119749, Mascot score 126, amino acid sequence in figure 2). The corresponding coding gene was located on the outbreak strain’s ESBL plasmid, the transfer of which into *E. coli* TOP10 resulted in the appearance of the respective peak in the recipient strain’s spectrum. The peak at m/z 10883 represents a 97 amino acids protein of unknown function (GenPept accession YP_002404855, Mascot score 3504, amino acid sequence in figure 2) derived from a 116 amino acids precursor by cleavage of a 19 amino acid signal peptide predicted by SignalP 4.0 (D = 0.67, D-cutoff = 0.57) [27]. The mature protein has a calculated molecular weight of 10881.5 Da and is predicted to reside in the bacterium’s periplasmatic space (PSORTb 3.0 Periplasmatic score = 9.83) [28]. The coding sequence resides on the outbreak strain’s chromosome, directly adjacent to genes of the *cus*/*sil* gene cluster, involved in heavy metal resistance [29]. The gene can be found in identical genomic context on the chromosomes of other *E. coli* (GenBank accession YP_002404855), *Enterobacter cloacae* (CP001918) and *Cronobacter sakazakii* (CP000783) strains as well as on plasmids from *E. coli* (DQ517526), *Salmonella enterica* (JN983042) and *Serratia marcescens* (BX664015).

Neither of the identified biomarker proteins was listed among the candidate proteins obtained by molecular weight matching because of incorrect annotation of the translation start (m/z 6711) or the signal peptide sequence (m/z 10883) in the employed databases.

In-silico cross-validation against NCBI’s refseq_genomic database confirmed the low occurrence rates predicted for both marker proteins from the analysis of locally acquired mass spectra. Alleles translating into proteins compatible with peaks at m/z 6711 and m/z 10883 were found in only 0.6% and 5.5% of the 162 *E. coli* plasmids and 55 chromosomes, present in the database as of July 2012.

### Mass spectrometry based strain typing

**Figure 3:**
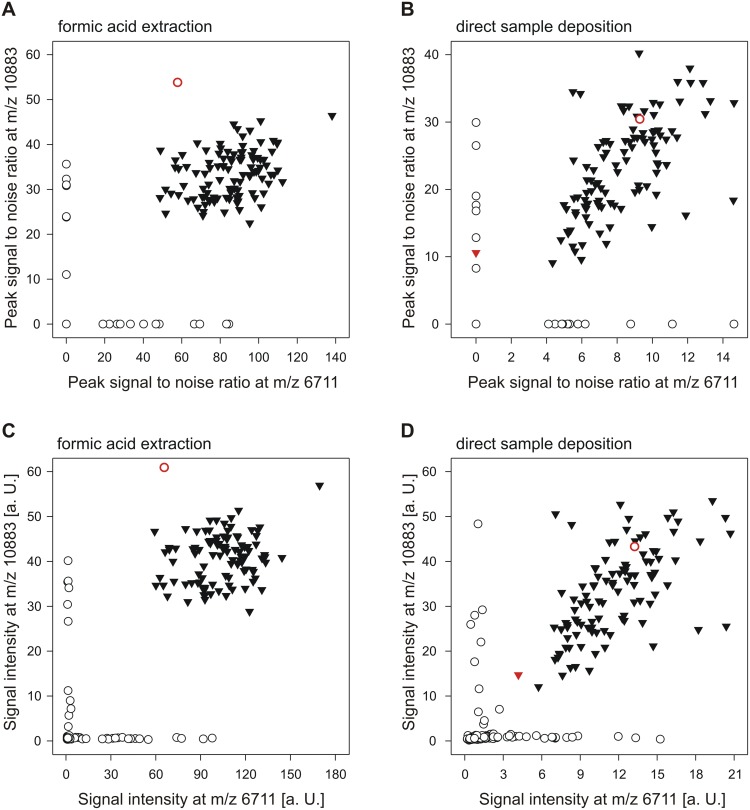
Marker peak characteristics in spectra from study isolates. Mean signal to noise ratio of outbreak strain marker peaks (A, B) and mean signal intensitiy (C, D) at marker peak position in formic acid extraction (A, C) and direct sample deposition (B, D) triplicate spectra from 293 study isolates. Black triangles and white circles represent measurements from 104 outbreak and 189 non outbreak *E. coli* isolates, respectively. Red colour indicates misidentified isolates.

The established MALDI-TOF MS typing scheme was evaluated with 293 clinical *E. coli* isolates (221 recovered from stool, 59 from urine and 13 from rectal swabs), 104 (35.5%) of which were recognized as outbreak related by PCR genotyping. Using formic acid extraction spectra, MALDI-TOF typing correctly classified 292 (99.7%) of the 293 study isolates, including all 104 outbreak related isolates (table 2). The observed signal intensities and signal to noise ratios for both marker peaks (figure 3) allowed for automated marker peak detection in all 312 outbreak isolate triplicate spectra (table 3). Likewise, absence of at least one of the marker peaks led to correct classification of 188 (99.5%) of the 189 non outbreak isolates. Rapid sample preparation by direct deposition, as performed by many clinical laboratories for routine species level identification, resulted in reduced signal intensity (figure 3) and peak detectability (table 4) for the m/z 6711 marker peak. Consequently, the overall correct classification rate dropped to 99.0%.

**Table 2:** Correct classification rates (%) of MALDI-TOF typing.

Sample preparation	OREC	NOREC
FAE	104/104 (100.0)	188/189 (99.5)
DSD	102/104 (98.1)	188/189 (99.5)

Abbreviations: OREC outbreak related *E. coli* isolates, NOREC non outbreak related *E. coli* isolates, FAE formic acid extraction, DSD direct sample deposition.

**Table 3:** Peak detection rates (%) among study isolates.

	m/z 6711		m/z 10883	
Isolate classification	FAE	DSD	FAE	DSD
OREC	104/104 (100.0)	103/104 (99.0)	104/104 (100.0)	104/104 (100.0)
NOREC	14^a^/189 (7.9)	11/189 (5.8)	8^a^/189 (4.2)	8/189 (4.2)

a Confirmed by visual spectrum inspection and sequencing of the corresponding marker protein gene.

Abbreviations: OREC outbreak related *E. coli* isolates, NOREC non outbreak related *E. coli* isolates, FAE formic acid extraction, DSD direct sample deposition.

**Table 4:** Distribution (%) of peak detection rates in triplicate spectra of outbreak isolates.

	m/z 6711		m/z 10883	
Peak detection rate	FAE	DSD	FAE	DSD
0 of 3	0 (0.0)	1 (1.0)	0 (0.0)	0 (0.0)
1 of 3	0 (0.0)	7 (6.7)	0 (0.0)	0 (0.0)
2 of 3	0 (0.0)	18 (17.3)	0 (0.0)	1 (1.0)
3 of 3	104 (100.0)	78 (75.0)	104 (100.0)	103 (99.0)

Abbreviations: FAE formic acid extraction, DSD direct sample deposition.

Only one isolate (Isolate ID 48653866) was repeatedly misclassified with both sample preparation techniques. While the MALDI-TOF detection of both outbreak strain marker proteins could be confirmed by PCR and allele sequencing, PCR genotyping (*stx2*, *aggC*, *terD* and *rfbO104* negative) and MLST (sequence type 10) clearly classified the isolate as non outbreak related. In addition to this misclassified strain, 14 other non outbreak study isolates tested positive for the m/z 6711 marker peak (table 3). The frequency of this peak among non outbreak study isolates (7.9%) thus markedly exceeded the value observed for pre-outbreak spectra (0.0%). Visual spectrum inspection and PCR testing confirmed biomarker presence in all marker peak positive isolates. Like the outbreak strain, these isolates exhibited an ESBL phenotype. The responsible plasmid could be transferred into *E. coli* TOP10 in nine cases, giving rise to the characteristic peak at m/z 6711. DraI and HindIII plasmid restriction patterns from these transformants were indistinguishable from TY-2482, suggesting transmission of the outbreak strain’s ESBL plasmid to resident isolates. Remarkably, patients carrying these m/z 6711 marker peak positive non outbreak isolates had the outbreak strain recovered from earlier stool samples.

The observed frequency of the m/z 10883 marker peak (representing a chromosomally encoded protein) was consistent with the analysis of pre-outbreak spectra (4.2% and 4.7%).

PCR testing for both marker protein genes demonstrated excellent correlation between genotype and MALDI-TOF phenotype. All 126 marker peak positive study isolates also tested positive for the respective gene. Likewise, all 40 PCR positive, marker peak negative isolates could be shown to harbor a variant of the corresponding gene encoding for a protein with differing molecular weight (table 5). Detailed mass spectrometry results for all study isolates are provided in table S1.

**Table 5:** Variants of marker peak protein genes found among non outbreak study isolates by PCR and sequencing.

Primer set^a^	Variant	Gene sequence [accession]	Predicted MW [Da]	Number of isolates
mp6711	mp6711_v1^b^	HF569077	6709.8	14
	mp6711_v2	HF569078	5706.6	2
	mp6711_v3	HF569079	5732.7	2
	mp6711_v4	HF569080	5750.6	4
	mp6711_v5	HF569081	6210.1	1
	mp6711_v6	HF569082	6224.2	3
	mp6711_v7	HF569083	6236.3	6
	mp6711_v8	HF569084	6697.7	8
	mp6711_v9	HF569085	7644.8	5
	unknown^c^	-	-	5
mp10883	mp10883_v1^b^	HF569086	10881.5	8
	mp10883_v2	HF569087	10863.4	3
	unknown^c^	-	-	2

a See table 1.

b Outbreak strain variant.

c No full length gene sequences for molecular weight prediction could be obtained from the respective isolates. Available partial sequences differ from the outbreak strain variant.

Abbreviations: MW Molecular weight.

### Isolate classification by whole spectrum similarity

With retrospectively chosen threshold values, whole spectrum similarity comparison to reference spectra yielded classification accuracies of at most 98% (table 6). The highest AUC values were obtained with simple binary distance measures (e.g. Jaccard) applied to formic acid extraction spectra. Unweighted metric distance measures (Euclidean, Manhattan) and standard Biotyper-scoring yielded significantly lower AUCs. Irrespective of the distance measure employed, analysis of formic acid extraction spectra resulted in better classification results as compared to direct sample deposition.

**Table 6:** Isolate classification with whole spectrum similarity measures.

		FAE		DSD	
Distance measure	Type	AUC	Accuracy	AUC	Accuracy
Jaccard	B	0.998	0.980	0.988^b^	0.942
Dice	B	0.998	0.980	0.988^b^	0.942
Kulczynski2	B	0.998	0.980	0.985^a^^,^ b	0.946
Mountford	B	0.998	0.980	0.987^b^	0.939
Braun-Blanquet	B	0.999	0.983	0.987^b^	0.946
Simpson	B	0.997	0.973	0.974^a^^,^ b	0.935
Ochiai	B	0.998	0.980	0.987^b^	0.939
Euclidean	M	0.972^a^	0.939	0.903^a^^,^ b	0.884
Bhjattacharyya	M	0.991	0.980	0.973^a^^,^ b	0.953
Divergence	m	0.997^a^	0.973	0.979^a^^,^ b	0.932
Manhattan	m	0.974^a^	0.929	0.923^a^^,^ b	0.898
Canberra	m	0.993^a^	0.966	0.967^a^^,^ b	0.922
Pearson	c	0.995	0.980	0.973^a^^,^ b	0.908
Biotyper 3.0	-	0.979^a^	0.932	0.877^a^^,^ b	0.823

a Significant difference to the Jaccard distance (p<0.05).

b Significant difference to FAE sample preparation (p<0.05).

Abbreviations: b binary, m metric, c correlation, FAE formic acid extraction, DSD direct sample deposition, AUC area under the ROC curve.

**Figure 4:**
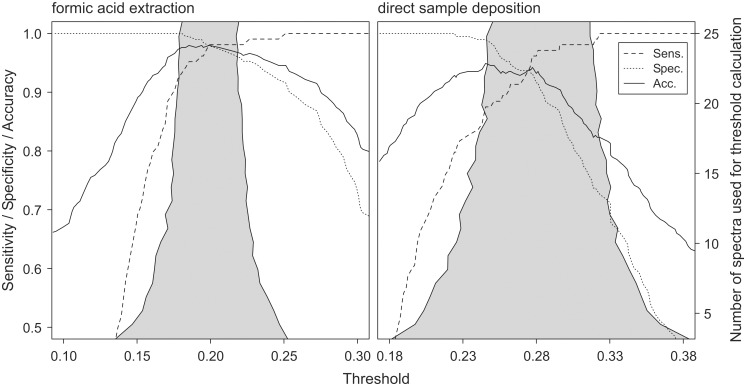
Performance of isolate classification by whole spectrum similarity to reference spectra. Accuracy, sensitivity and specificity for the classification of study isolates by Jaccard’s distance to TY-2482 reference spectra as a function of the selected threshold. Grey areas represent bootstrap estimates of 95% confidence intervals for thresholds derived from the distribution of distance values among outbreak isolate triplicate spectra.

Within the 95% confidence interval for a threshold prospectively set by the analysis of 25 outbreak strain triplicate spectra, sensitivity and specificity of isolate classification with Jaccard’s distance varied from 92 to 98% and 95 to 100% for formic acid extraction spectra and from 88 to 98% and 75 to 98% for direct sample deposition spectra, respectively (figure 4).

### Spectral variability among endemic *E. coli* isolates

**Figure 5:**
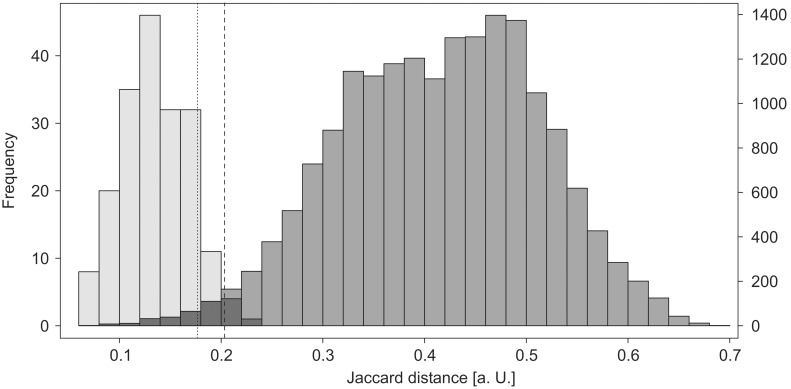
Whole spectrum similarity among non outbreak study isolates. Distribution of Jaccard distance values from pairwise spectrum comparisons among non outbreak study isolates (dark grey, n = 17955) and single isolate replicate spectra (light grey, n = 189). The dashed line represents a threshold for spectral identity derived from the replicate spectrum distribution (mean+2×SD). The dotted line represents a less conservative threshold that would correctly classify 95% of all isolate pairs that were found spectrally identical upon manual spectrum comparison.

Overall spectral variability among endemic *E. coli* strains was estimated from 17955 pairwise whole spectrum similarity comparisons between non outbreak study isolates (figure 5). Only 282 (1.6%) isolate pairs were classified as spectrally identical using a distance threshold derived from the normal distribution of distance values for comparisons between replicate spectra (mean = 0.136, SD = 0.034, Shapiro Wilk W(189) = 0.990, p = 0.24). Whole spectrum similarity distance values were found to be in good correlation with manual spectrum comparison (point biserial correlation coefficient = 0.62, p≤0.0001), which suggested an even lower proportion of identical isolate pairs (74, 0.4%). Simpson’s diversity index for hierarchically clustered spectral distance values was below 0.01, indicating a high degree of spectral variability among endemic *E. coli* isolates.

#### Spectrum data

Spectrum data is available from the Dryad Digital Repository: http://doi.org/10.5061/dryad.bq64j.

## Discussion

Whole cell MALDI-TOF mass spectrometry has replaced biochemical profiling as method of choice for species level identification of cultured microorganisms. The technique’s superior operational characteristics have also generated considerable interest in application for epidemiological purpose. Subspecies differentiation by the analysis of whole cell MALDI-TOF mass spectra has so far been performed in a number of taxonomic studies to support single- or multilocus sequencing based phylogenies [30]–[32]. Applications in medical microbiology encompass the biomarker based identification of typhoid *Salmonella enterica* [33] and several epidemiological proof-of-concept studies. Further implementation into clinical laboratories has so far been impeded by the lack of standardized workflows, dedicated software tools and publicly accessible spectrum collections for in-silico development and validation of novel typing strategies.

The present study demonstrates the successful use of a general applicable biomarker based MALDI-TOF typing strategy during a large STEC outbreak. In contrast to previous approaches, biomarker discovery did not involve cumbersome de-novo spectrum acquisition from purpose built reference strain collections [11]–[12] but completely relied upon spectra which had already been collected at for routine species identification. Corresponding data is readily available to a growing number of laboratories performing MALDI-TOF MS fingerprinting as part of their routine pathogen identification workflow and should facilitate application of the presented strategy to outbreak situation involving different strains and species.

Molecular identification of biomarker candidates allowed for in-silico cross-validation of the mass spectrometry typing scheme against existing nucleic acid and protein databases and facilitated the confirmation of mass spectrometry results by PCR. Knowledge of the protein behind the peak also provided the key clue to explain unexpected peak frequencies among non-outbreak related isolates as a result of plasmid transmission. Compared to simple molecular weight matching, the use of tandem mass spectrometry for biomarker protein identification in a top-down proteomics approach offers better specificity and is much less likely to produce ambiguous results [34].

The performance of mass spectrometry based typing for the identification of STEC outbreak isolates was similar to established nucleic acid based strategies. The combination of two independent marker peaks ensured a low false positive rate despite sporadic transmission of the plasmid encoded biomarker peak to endemic strains. Replicate measurements compensated for the reduction in signal quality associated with the widely used direct sample deposition method and facilitated the integration of mass spectrometry based typing into an existing pathogen identification workflow.

The marker peak based approach provided better classification results than whole spectrum similarity comparisons to reference spectra and was more robust with respect to the lower quality of direct sample deposition spectra.

As only a small subset of the microbial proteome (about 1%) is represented in whole cell MALDI-TOF spectra [35], the technique cannot, on principle, achieve the phylogenetic resolution of genome wide nucleic acid based typing strategies [36]. However, at least for *E. coli*, results from the analysis of spectral variability among endemic isolates suggest sufficient discriminatory power for epidemiological purpose.

In contrast to nucleic acid sequences or PFGE-patterns, spectra for MS typing can be acquired at negligible additional costs as part of the routine pathogen identification workflow [37]. Given the accumulating evidence, that the technique provides sufficient discriminatory power for routine typing tasks, MALDI-TOF MS could facilitate real-time outbreak surveillance.

## Supporting Information

Table S1 **Mass spectrometry results for all study isolates.** The column ‘class’ notes the reference classification of an isolate as outbreak related (orec) or non-outbreak related (norec). Columns ‘orecPCR’ and ‘orecMS’ note the classification of an isolate as outbreak related by PCR and mass spectrometry, respectively. Columns ‘p6711’, ‘p10883’ and ‘p10300’ note the detection of a peak at the respective mz-position. Columns ‘maxint6711’, ‘maxint 10883’ and ‘maxint10300’ show the highest signal intensity in a 400 ppm window around the respective mz-position. Columns ‘p6711mz’, ‘p10883mz’ and ‘p10300mz’ show the exact mz position of the detected peak. ‘Columns p6711snr’, ‘p10883snr’ and ‘p10300snr’ show the signal to noise ration of the peak detected at the respective mz-position. Columns ‘p6711int’, ‘p10883int’ and ‘p10300int’ show the signal intensity of the peak detected at the respective mz-position. Columns ‘meanMz’, ‘meanSnr’ and ‘meanInt’ show the mean values for three technical replicates. Prefixes ‘dsd_’ and ‘fae_’ indicate spectrum acquisition by direct sample deposition and formic acid extraction, respectively.
(TXT)

## References

[1] Seng P, Drancourt M, Gouriet F, La Scola B, Fournier PE, et al (2009) Ongoing revolution in bacteriology: routine identification of bacteria by matrix-assisted laser desorption ionization time-of-flight mass spectrometry. Clin Infect Dis 49: 543–551. 19583519 10.1086/600885

[2] Holland RD, Wilkes JG, Rafii F, Sutherland JB, Persons CC, et al (1996) Rapid identification of intact whole bacteria based on spectral patterns using matrix-assisted laser desorption/ionization with time-of-flight mass spectrometry. Rapid Commun Mass Spectrom 10: 1227–1232. 8759332 10.1002/(SICI)1097-0231(19960731)10:10<1227::AID-RCM659>3.0.CO;2-6

[3] Sauer S, Freiwald A, Maier T, Kube M, Reinhardt R, et al (2008) Classification and identification of bacteria by mass spectrometry and computational analysis. PLoS One 3: e2843. 18665227 10.1371/journal.pone.0002843 PMC2475672

[4] Barbuddhe SB, Maier T, Schwarz G, Kostrzewa M, Hof H, et al (2008) Rapid identification and typing of listeria species by matrix-assisted laser desorption ionization-time of flight mass spectrometry. Appl Environ Microbiol 74: 5402–5407. 18606788 10.1128/AEM.02689-07 PMC2546641

[5] Friedrichs C, Rodloff AC, Chhatwal GS, Schellenberger W, Eschrich K (2007) Rapid identification of viridans streptococci by mass spectrometric discrimination. J Clin Microbiol 45: 2392–2397. 17553974 10.1128/JCM.00556-07 PMC1951256

[6] Grosse-Herrenthey A, Maier T, Gessler F, Schaumann R, Bohnel H, et al (2008) Challenging the problem of clostridial identification with matrix-assisted laser desorption and ionization-time-of-flight mass spectrometry (MALDI-TOF MS). Anaerobe 14: 242–249. 18621134 10.1016/j.anaerobe.2008.06.002

[7] Stephan R, Cernela N, Ziegler D, Pfluger V, Tonolla M, et al (2011) Rapid species specific identification and subtyping of *Yersinia enterocolitica* by MALDI-TOF mass spectrometry. J Microbiol Methods 87: 150–153. 21914454 10.1016/j.mimet.2011.08.016

[8] Karger A, Ziller M, Bettin B, Mintel B, Schares S, et al (2011) Determination of serotypes of Shiga toxin-producing *Escherichia coli* isolates by intact cell matrix-assisted laser desorption ionization-time of flight mass spectrometry. Appl Environ Microbiol 77: 896–905. 21115707 10.1128/AEM.01686-10 PMC3028713

[9] Dieckmann R, Malorny B (2011) Rapid screening of epidemiologically important *Salmonella enterica* subsp. enterica serovars by whole-cell matrix-assisted laser desorption ionization-time of flight mass spectrometry. Appl Environ Microbiol 77: 4136–4146. 21515723 10.1128/AEM.02418-10 PMC3131644

[10] Reil M, Erhard M, Kuijper EJ, Kist M, Zaiss H, et al (2011) Recognition of *Clostridium difficile* PCR-ribotypes 001, 027 and 126/078 using an extended MALDI-TOF MS system. Eur J Clin Microbiol Infect Dis 30: 1431–1436. 21503840 10.1007/s10096-011-1238-6 PMC3191295

[11] Wolters M, Rohde H, Maier T, Belmar-Campos C, Franke G, et al (2011) MALDI-TOF MS fingerprinting allows for discrimination of major methicillin-resistant *Staphylococcus aureus* lineages. Int J Med Microbiol 301: 64–68. 20728405 10.1016/j.ijmm.2010.06.002

[12] Boggs SR, Cazares LH, Drake R (2012) Characterization of a *Staphylococcus aureus* USA300 protein signature using matrix-assisted laser desorption/ionization time-of-flight mass spectrometry. J Med Microbiol 61: 640–644. 22322338 10.1099/jmm.0.037978-0

[13] Frank C, Werber D, Cramer JP, Askar M, Faber M, et al (2011) Epidemic profile of Shiga-toxin-producing *Escherichia coli* O104:H4 outbreak in Germany. N Engl J Med 365: 1771–1780. 21696328 10.1056/NEJMoa1106483

[14] Rohde H, Qin J, Cui Y, Li D, Loman NJ, et al (2011) Open-source genomic analysis of Shiga-toxin-producing *E. coli* O104:H4. N Engl J Med 365: 718–724. 21793736 10.1056/NEJMoa1107643

[15] Freiwald A, Sauer S (2009) Phylogenetic classification and identification of bacteria by mass spectrometry. Nat Protoc 4: 732–742. 19390529 10.1038/nprot.2009.37

[16] Gibb S, Strimmer K (2012) MALDIquant: a versatile R package for the analysis of mass spectrometry data. Bioinformatics 28: 2270–2271. 22796955 10.1093/bioinformatics/bts447

[17] R Development Core Team (2008) R: A Language and Environment for Statistical Computing. Vienna, Austria.

[18] Gasteiger E, Hoogland C, Gattiker A, Duvaud S, Wilkins MR, et al.. (2005) Protein Identification and Analysis Tools on the ExPASy Server. In: Walker JM, editor. The Proteomics Protocols Handbook: Humana Press. 571–607.

[19] Perkins DN, Pappin DJ, Creasy DM, Cottrell JS (1999) Probability-based protein identification by searching sequence databases using mass spectrometry data. Electrophoresis 20: 3551–3567. 10612281 10.1002/(SICI)1522-2683(19991201)20:18<3551::AID-ELPS3551>3.0.CO;2-2

[20] Pruitt KD, Tatusova T, Klimke W, Maglott DR (2009) NCBI Reference Sequences: current status, policy and new initiatives. Nucleic Acids Res 37: D32–36. 18927115 10.1093/nar/gkn721 PMC2686572

[21] Mellmann A, Harmsen D, Cummings CA, Zentz EB, Leopold SR, et al (2011) Prospective genomic characterization of the German enterohemorrhagic *Escherichia coli* O104:H4 outbreak by rapid next generation sequencing technology. PLoS One 6: e22751. 21799941 10.1371/journal.pone.0022751 PMC3140518

[22] Bielaszewska M, Mellmann A, Zhang W, Kock R, Fruth A, et al (2011) Characterisation of the *Escherichia coli* strain associated with an outbreak of haemolytic uraemic syndrome in Germany, 2011: a microbiological study. Lancet Infect Dis 11: 671–676. 21703928 10.1016/S1473-3099(11)70165-7

[23] Qin J, Cui Y, Zhao X, Rohde H, Liang T, et al (2011) Identification of the Shiga toxin-producing *Escherichia coli* O104:H4 strain responsible for a food poisoning outbreak in Germany by PCR. J Clin Microbiol 49: 3439–3440. 21752971 10.1128/JCM.01312-11 PMC3165629

[24] Bielaszewska M, Kock R, Friedrich AW, von Eiff C, Zimmerhackl LB, et al (2007) Shiga toxin-mediated hemolytic uremic syndrome: time to change the diagnostic paradigm? PLoS One 2: e1024. 17925872 10.1371/journal.pone.0001024 PMC1995754

[25] Wirth T, Falush D, Lan R, Colles F, Mensa P, et al (2006) Sex and virulence in *Escherichia coli*: an evolutionary perspective. Mol Microbiol 60: 1136–1151. 16689791 10.1111/j.1365-2958.2006.05172.x PMC1557465

[26] Robin X, Turck N, Hainard A, Tiberti N, Lisacek F, et al (2011) pROC: an open-source package for R and S+ to analyze and compare ROC curves. BMC Bioinformatics 12: 77. 21414208 10.1186/1471-2105-12-77 PMC3068975

[27] Petersen TN, Brunak S, von Heijne G, Nielsen H (2011) SignalP 4.0: discriminating signal peptides from transmembrane regions. Nat Methods 8: 785–786. 21959131 10.1038/nmeth.1701

[28] Yu NY, Wagner JR, Laird MR, Melli G, Rey S, et al (2010) PSORTb 3.0: improved protein subcellular localization prediction with refined localization subcategories and predictive capabilities for all prokaryotes. Bioinformatics 26: 1608–1615. 20472543 10.1093/bioinformatics/btq249 PMC2887053

[29] Franke S, Grass G, Rensing C, Nies DH (2003) Molecular analysis of the copper-transporting efflux system CusCFBA of *Escherichia coli* . J Bacteriol 185: 3804–3812. 12813074 10.1128/JB.185.13.3804-3812.2003 PMC161567

[30] Sato H, Teramoto K, Ishii Y, Watanabe K, Benno Y (2011) Ribosomal protein profiling by matrix-assisted laser desorption/ionization time-of-flight mass spectrometry for phylogenety-based subspecies resolution of *Bifidobacterium longum* . Syst Appl Microbiol 34: 76–80. 20828962 10.1016/j.syapm.2010.07.003

[31] Munoz R, Lopez-Lopez A, Urdiain M, Moore ER, Rossello-Mora R (2011) Evaluation of matrix-assisted laser desorption ionization-time of flight whole cell profiles for assessing the cultivable diversity of aerobic and moderately halophilic prokaryotes thriving in solar saltern sediments. Syst Appl Microbiol 34: 69–75. 21242046 10.1016/j.syapm.2010.11.012

[32] Mulet M, Gomila M, Lemaitre B, Lalucat J, Garcia-Valdes E (2012) Taxonomic characterisation of Pseudomonas strain L48 and formal proposal of *Pseudomonas entomophila* sp. nov. Syst Appl Microbiol 35: 145–149. 22326814 10.1016/j.syapm.2011.12.003

[33] Kuhns M, Zautner AE, Rabsch W, Zimmermann O, Weig M, et al (2012) Rapid Discrimination of *Salmonella enterica* Serovar Typhi from Other Serovars by MALDI-TOF Mass Spectrometry. PLoS One 7: e40004. 22768195 10.1371/journal.pone.0040004 PMC3386914

[34] Fagerquist CK, Garbus BR, Miller WG, Williams KE, Yee E, et al (2010) Rapid identification of protein biomarkers of *Escherichia coli* O157:H7 by matrix-assisted laser desorption ionization-time-of-flight-time-of-flight mass spectrometry and top-down proteomics. Anal Chem 82: 2717–2725. 20232878 10.1021/ac902455d

[35] Ryzhov V, Fenselau C (2001) Characterization of the protein subset desorbed by MALDI from whole bacterial cells. Anal Chem 73: 746–750. 11248887 10.1021/ac0008791

[36] Pritchard L, Holden NJ, Bielaszewska M, Karch H, Toth IK (2012) Alignment-free design of highly discriminatory diagnostic primer sets for *Escherichia coli* O104:H4 outbreak strains. PLoS One 7: e34498. 22496820 10.1371/journal.pone.0034498 PMC3320637

[37] Griffin PM, Price GR, Schooneveldt JM, Schlebusch S, Tilse MH, et al (2012) Use of matrix-assisted laser desorption ionization-time of flight mass spectrometry to identify vancomycin-resistant enterococci and investigate the epidemiology of an outbreak. J Clin Microbiol 50: 2918–2931. 22740710 10.1128/JCM.01000-12 PMC3421795

